# Supplementary material for: A Pan-RNase Inhibitor Enabling CRISPR-mRNA Platforms for Engineering of Primary Human Monocytes
Source: Int J Mol Sci. 2022 Aug 28;23(17):9749. doi: 10.3390/ijms23179749 (PMC9456164; doi:10.3390/ijms23179749)
Supplement: Supplementary file 1 [file ijms-23-09749-s001.zip › ijms-1871605-supplementary.pptx]

## Slide 1
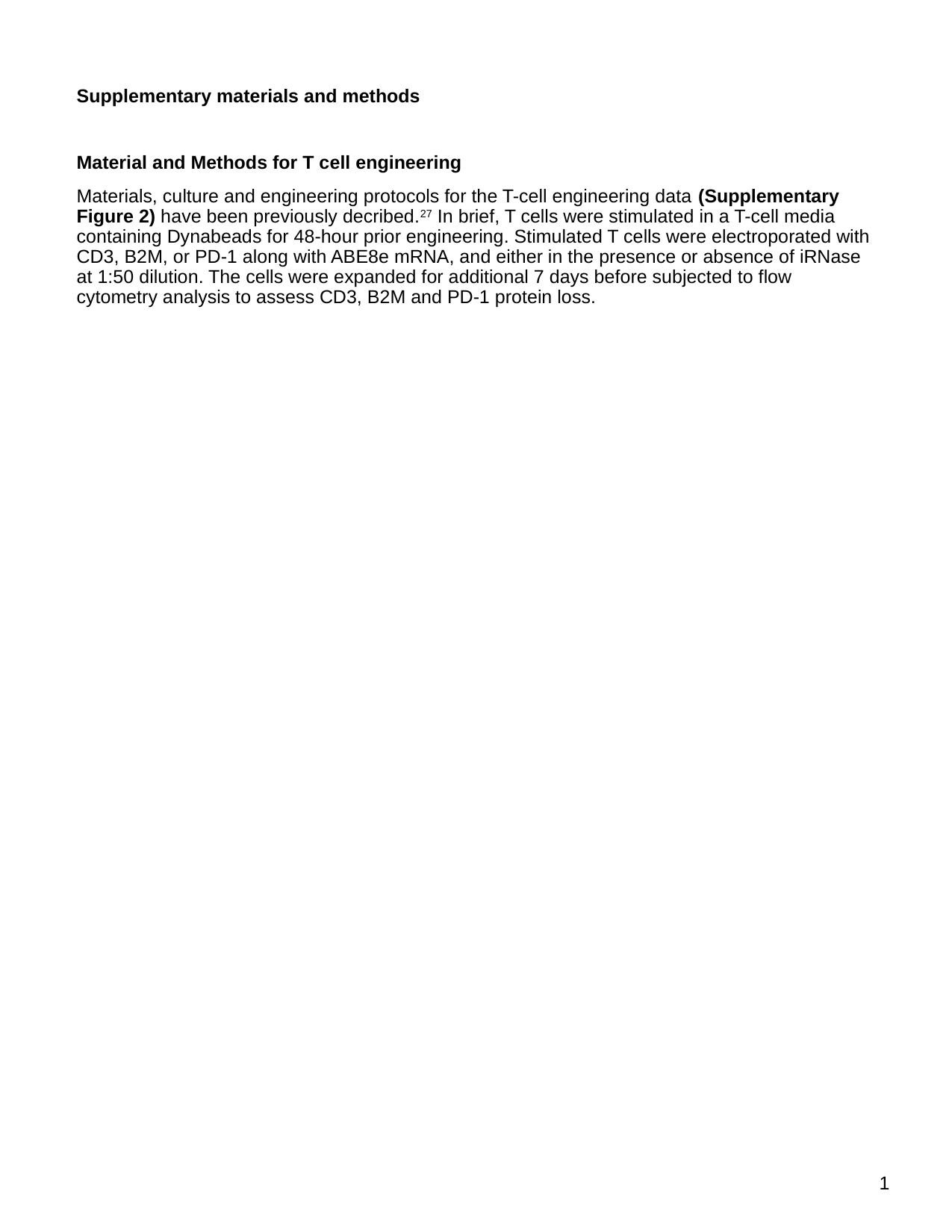

Supplementary materials and methods
Material and Methods for T cell engineering
Materials, culture and engineering protocols for the T-cell engineering data (Supplementary Figure 2) have been previously decribed.27 In brief, T cells were stimulated in a T-cell media containing Dynabeads for 48-hour prior engineering. Stimulated T cells were electroporated with CD3, B2M, or PD-1 along with ABE8e mRNA, and either in the presence or absence of iRNase at 1:50 dilution. The cells were expanded for additional 7 days before subjected to flow cytometry analysis to assess CD3, B2M and PD-1 protein loss.
1

## Slide 2
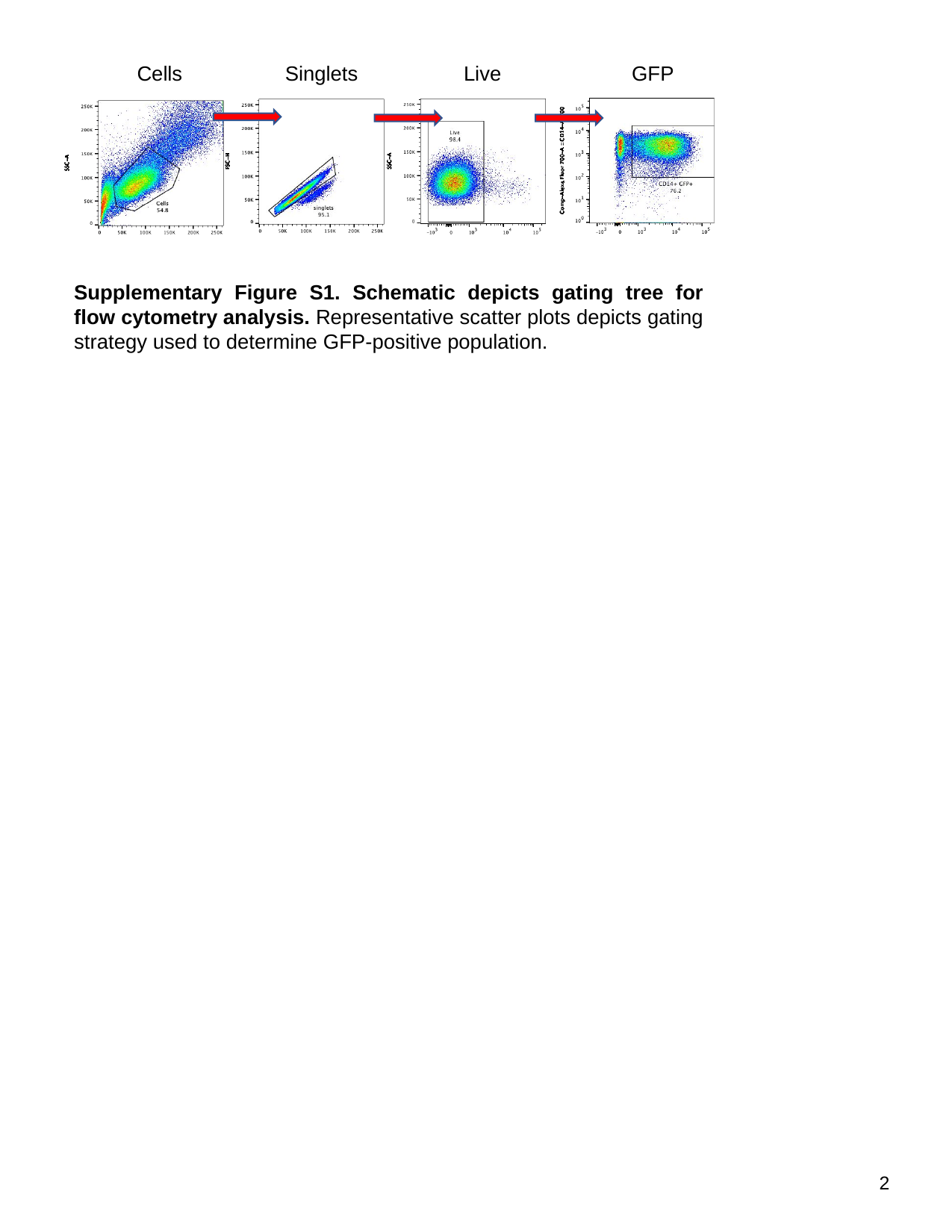

Live
GFP
Cells
Singlets
Supplementary Figure S1. Schematic depicts gating tree for flow cytometry analysis. Representative scatter plots depicts gating strategy used to determine GFP-positive population.
2

## Slide 3
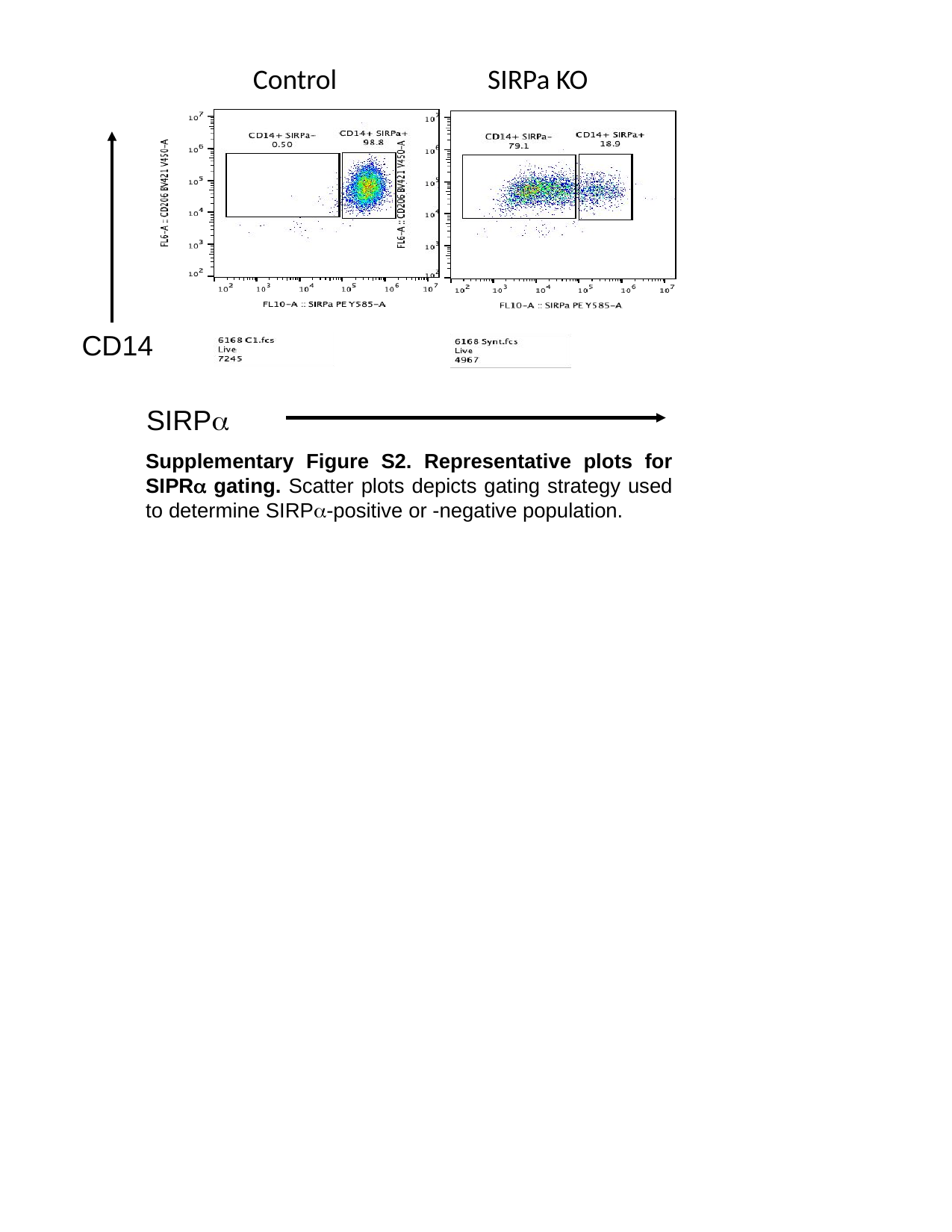

Control
SIRPa KO
CD14
SIRPa
Supplementary Figure S2. Representative plots for SIPRa gating. Scatter plots depicts gating strategy used to determine SIRPa-positive or -negative population.

## Slide 4
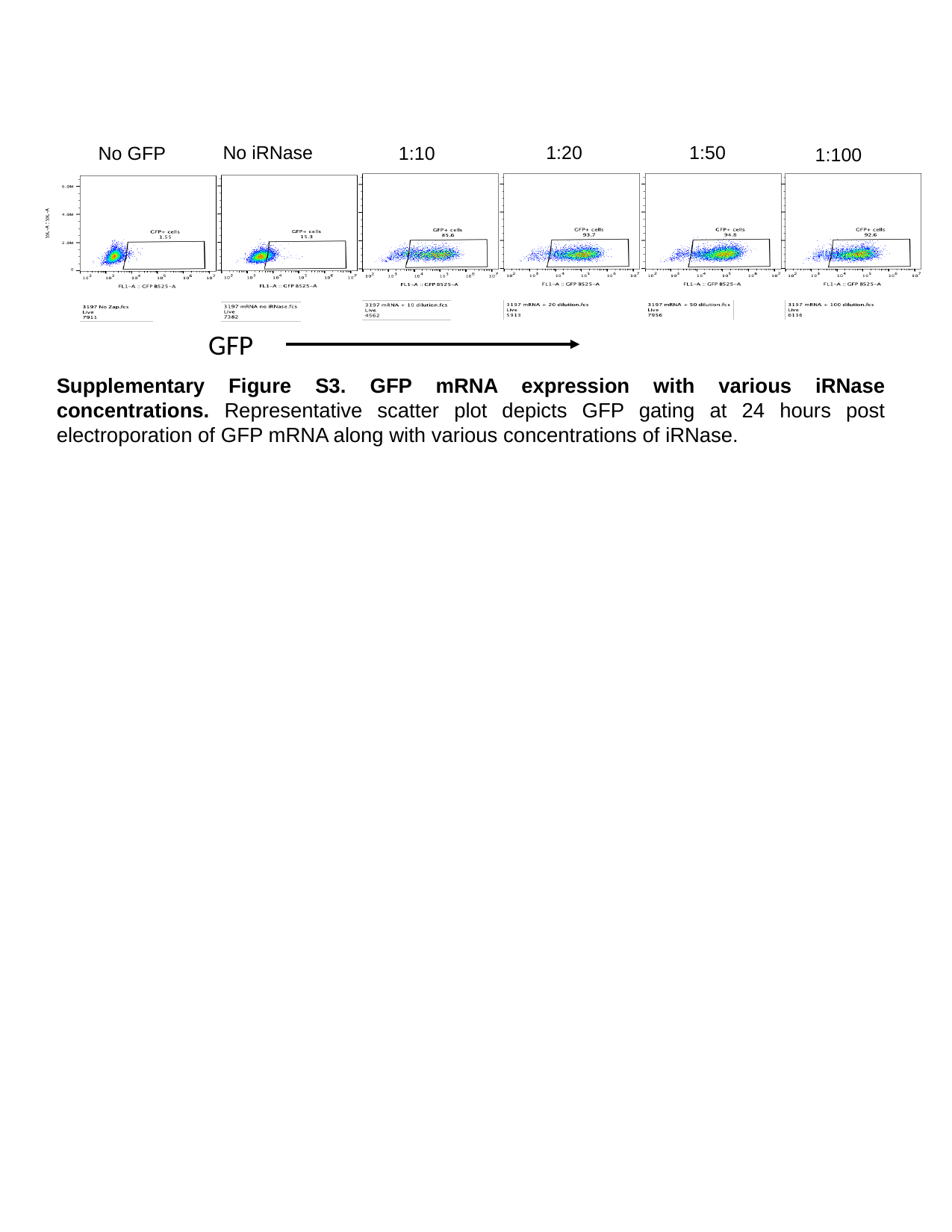

No iRNase
1:20
1:50
No GFP
1:10
1:100
GFP
Supplementary Figure S3. GFP mRNA expression with various iRNase concentrations. Representative scatter plot depicts GFP gating at 24 hours post electroporation of GFP mRNA along with various concentrations of iRNase.

## Slide 5
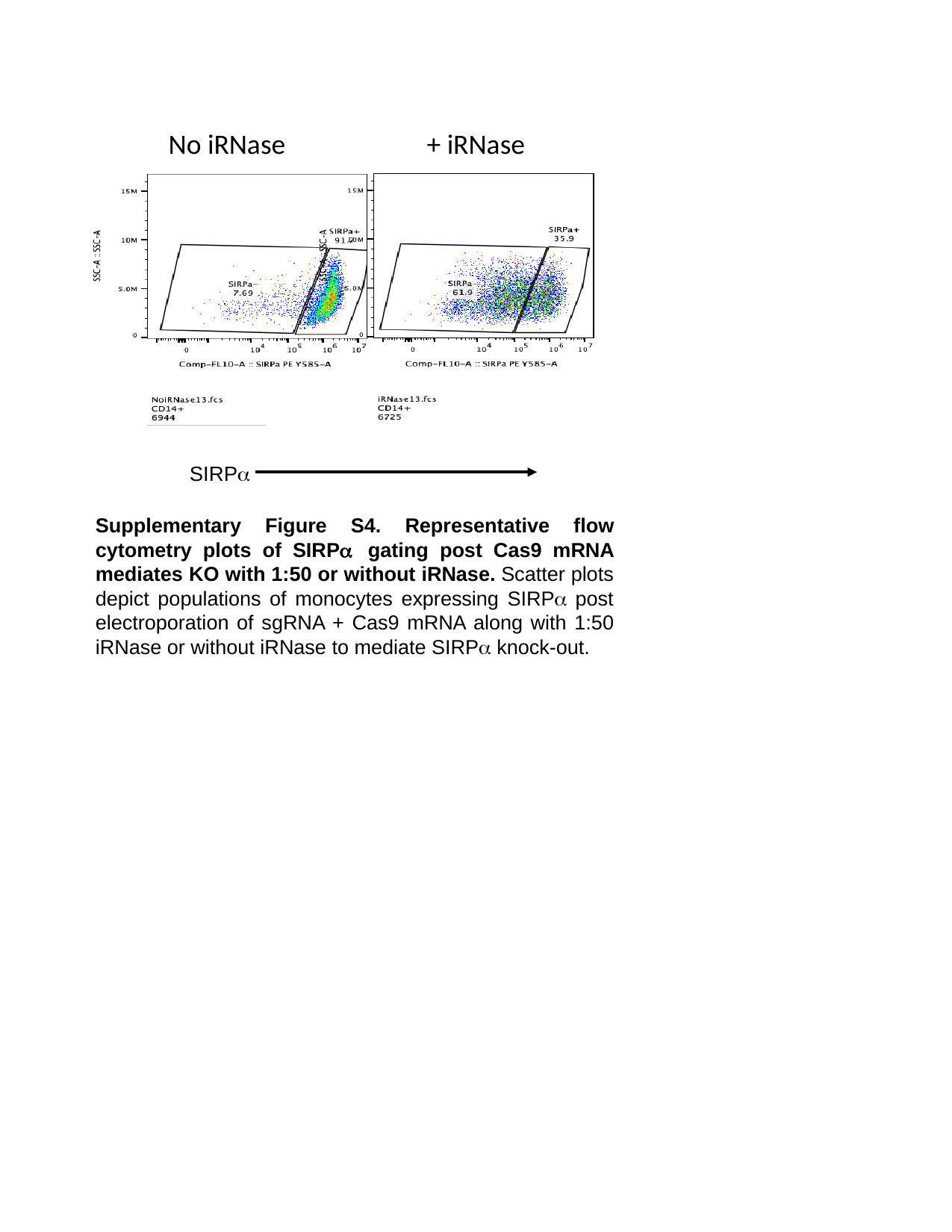

+ iRNase
No iRNase
SIRPa
Supplementary Figure S4. Representative flow cytometry plots of SIRPa gating post Cas9 mRNA mediates KO with 1:50 or without iRNase. Scatter plots depict populations of monocytes expressing SIRPa post electroporation of sgRNA + Cas9 mRNA along with 1:50 iRNase or without iRNase to mediate SIRPa knock-out.

## Slide 6
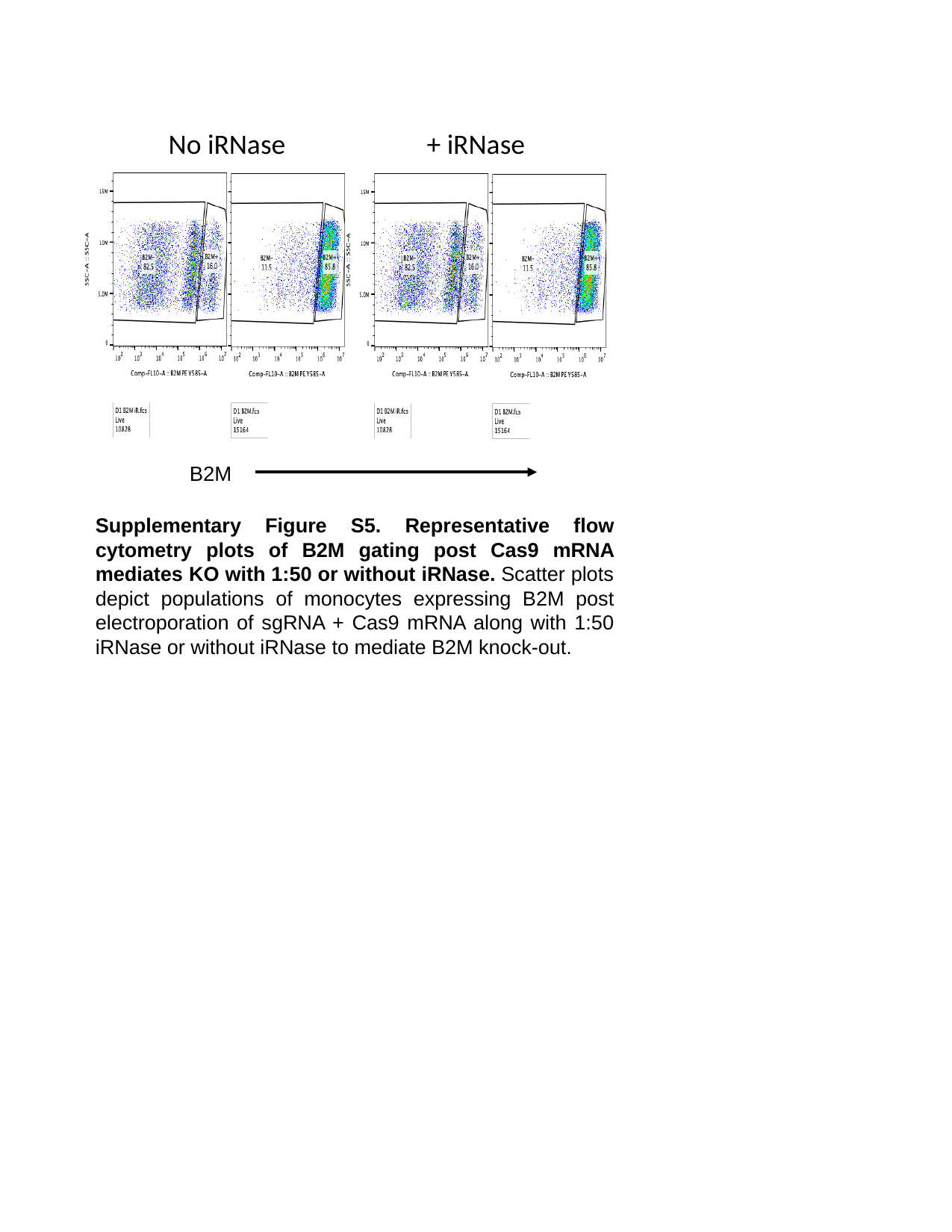

+ iRNase
No iRNase
B2M
Supplementary Figure S5. Representative flow cytometry plots of B2M gating post Cas9 mRNA mediates KO with 1:50 or without iRNase. Scatter plots depict populations of monocytes expressing B2M post electroporation of sgRNA + Cas9 mRNA along with 1:50 iRNase or without iRNase to mediate B2M knock-out.

## Slide 7
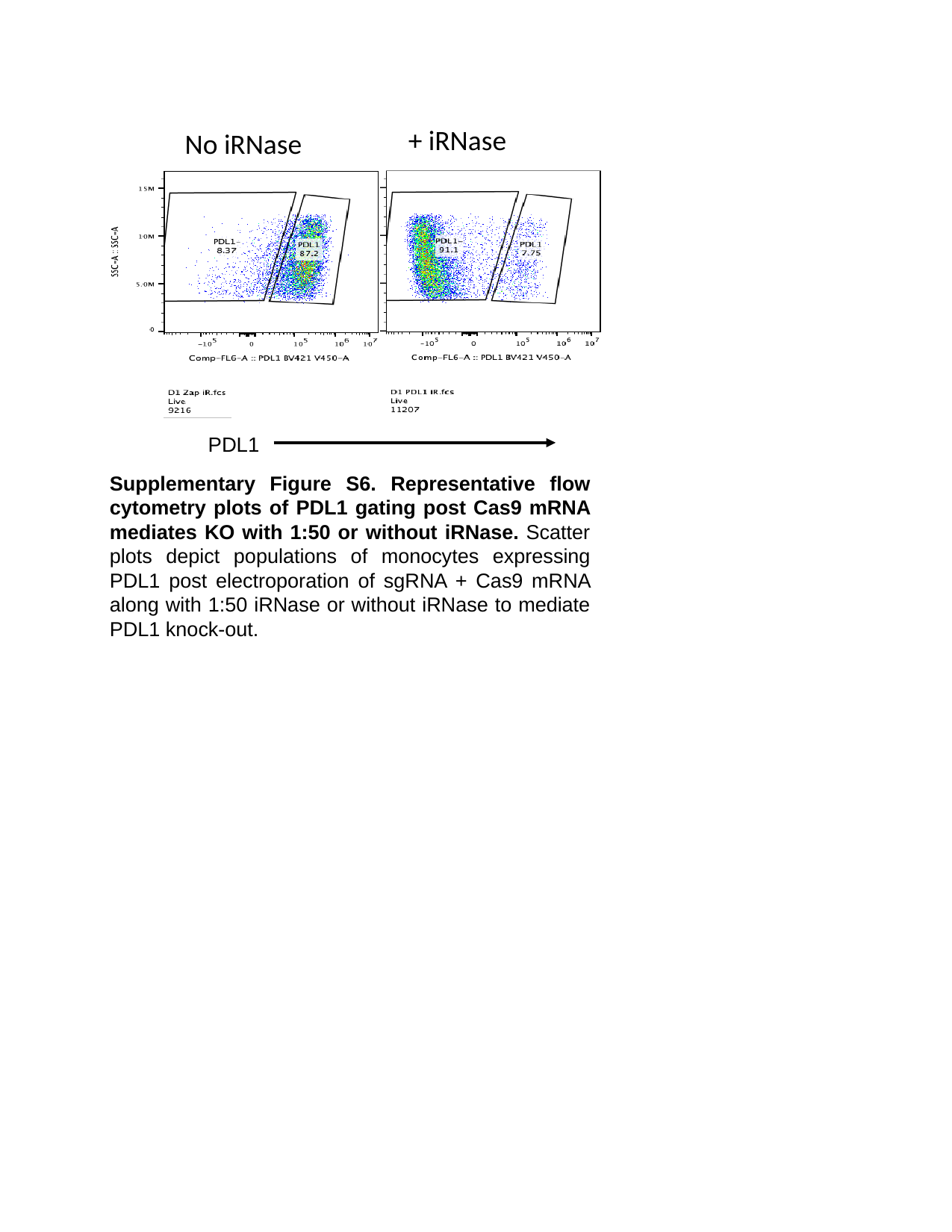

+ iRNase
No iRNase
PDL1
Supplementary Figure S6. Representative flow cytometry plots of PDL1 gating post Cas9 mRNA mediates KO with 1:50 or without iRNase. Scatter plots depict populations of monocytes expressing PDL1 post electroporation of sgRNA + Cas9 mRNA along with 1:50 iRNase or without iRNase to mediate PDL1 knock-out.

## Slide 8
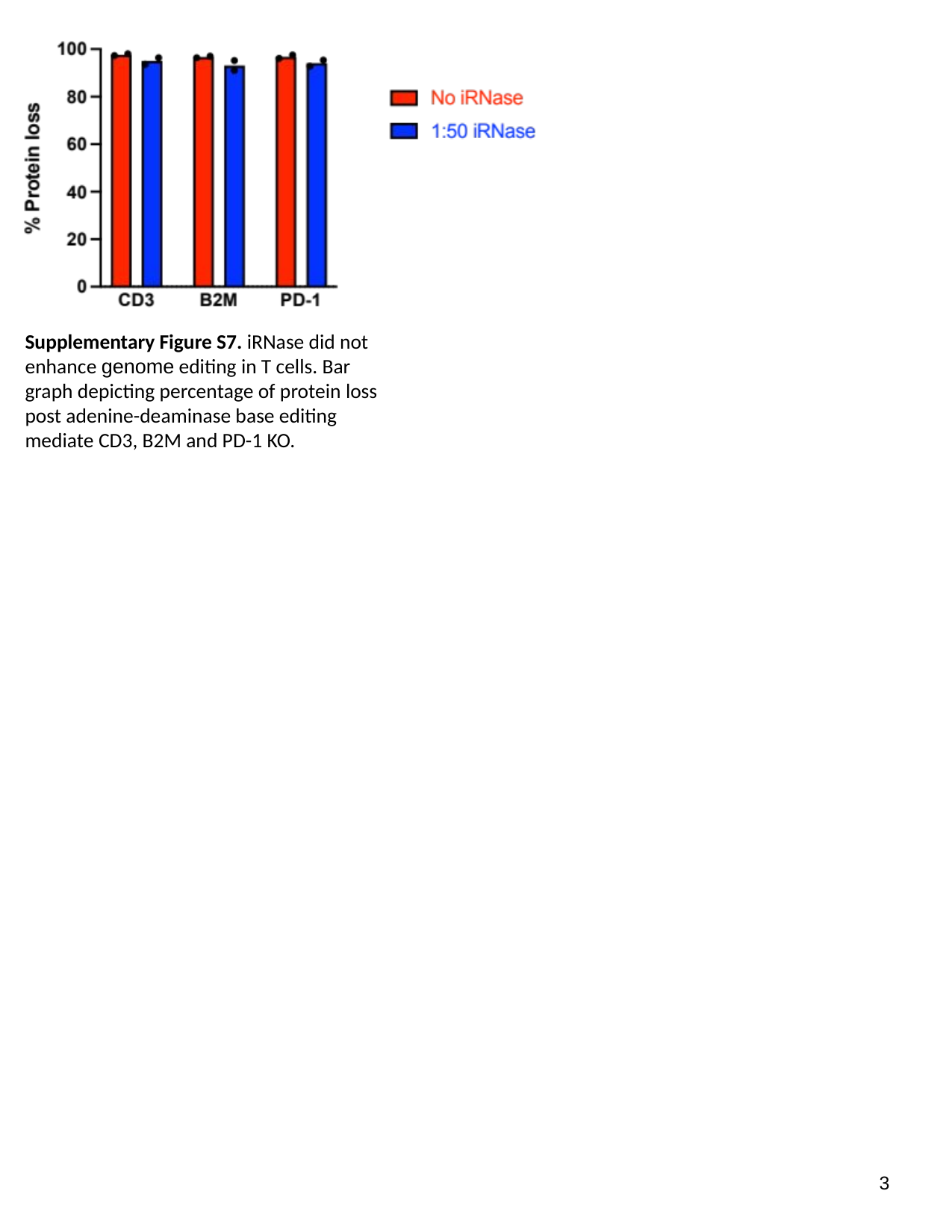

Supplementary Figure S7. iRNase did not enhance genome editing in T cells. Bar graph depicting percentage of protein loss post adenine-deaminase base editing mediate CD3, B2M and PD-1 KO.
3

## Slide 9
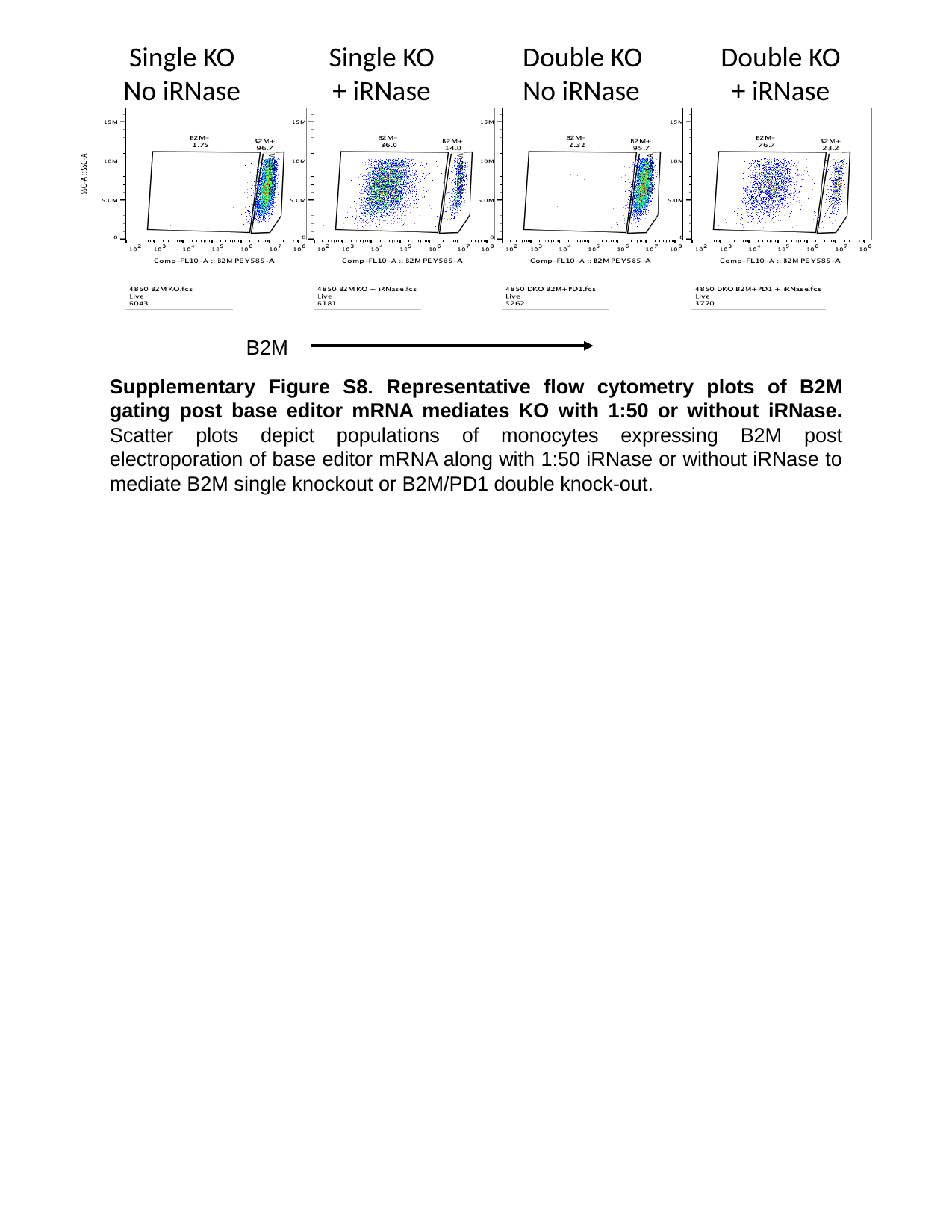

Single KO
+ iRNase
Double KO + iRNase
Double KO
No iRNase
Single KO
No iRNase
B2M
Supplementary Figure S8. Representative flow cytometry plots of B2M gating post base editor mRNA mediates KO with 1:50 or without iRNase. Scatter plots depict populations of monocytes expressing B2M post electroporation of base editor mRNA along with 1:50 iRNase or without iRNase to mediate B2M single knockout or B2M/PD1 double knock-out.

## Slide 10
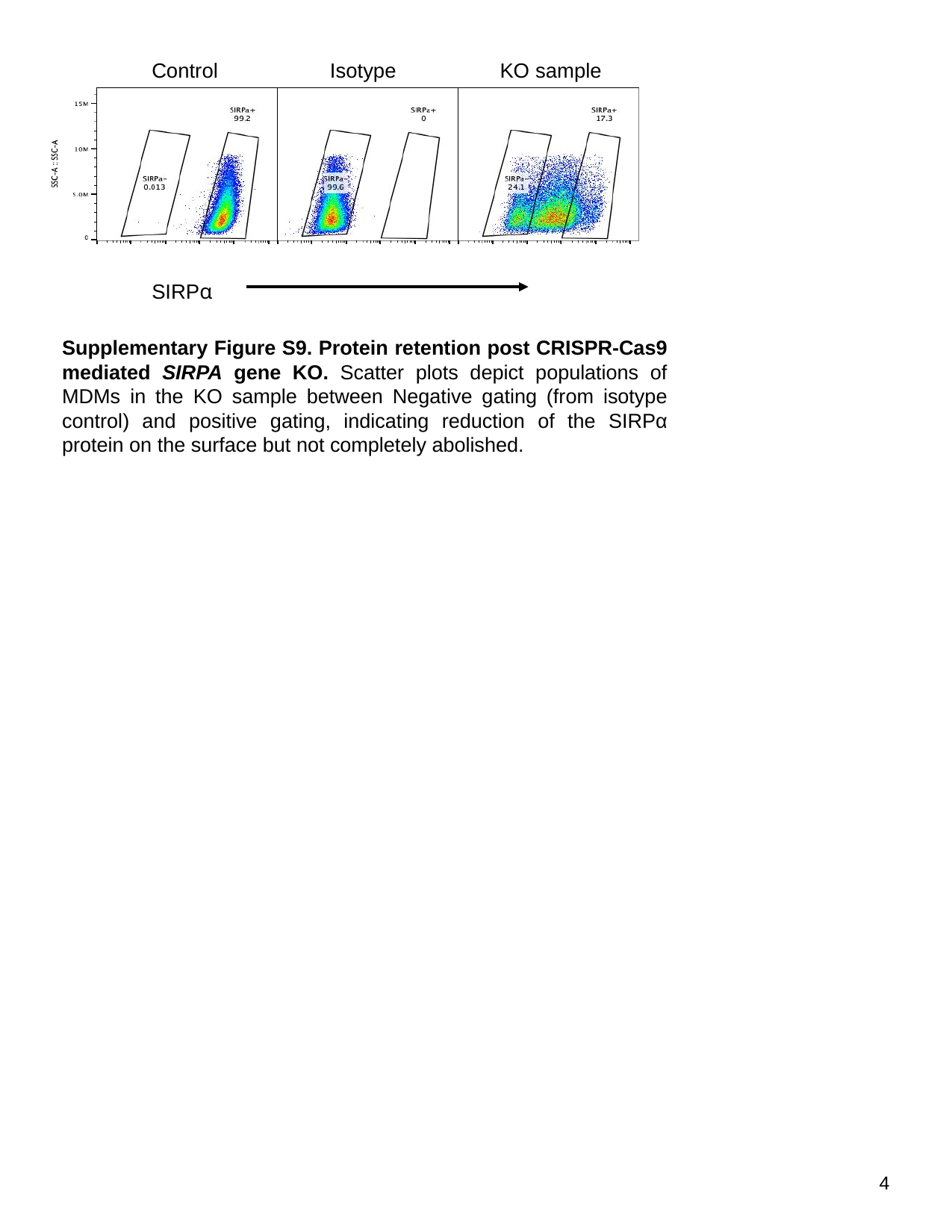

Control
Isotype
KO sample
SIRPα
Supplementary Figure S9. Protein retention post CRISPR-Cas9 mediated SIRPA gene KO. Scatter plots depict populations of MDMs in the KO sample between Negative gating (from isotype control) and positive gating, indicating reduction of the SIRPα protein on the surface but not completely abolished.
4

## Slide 11
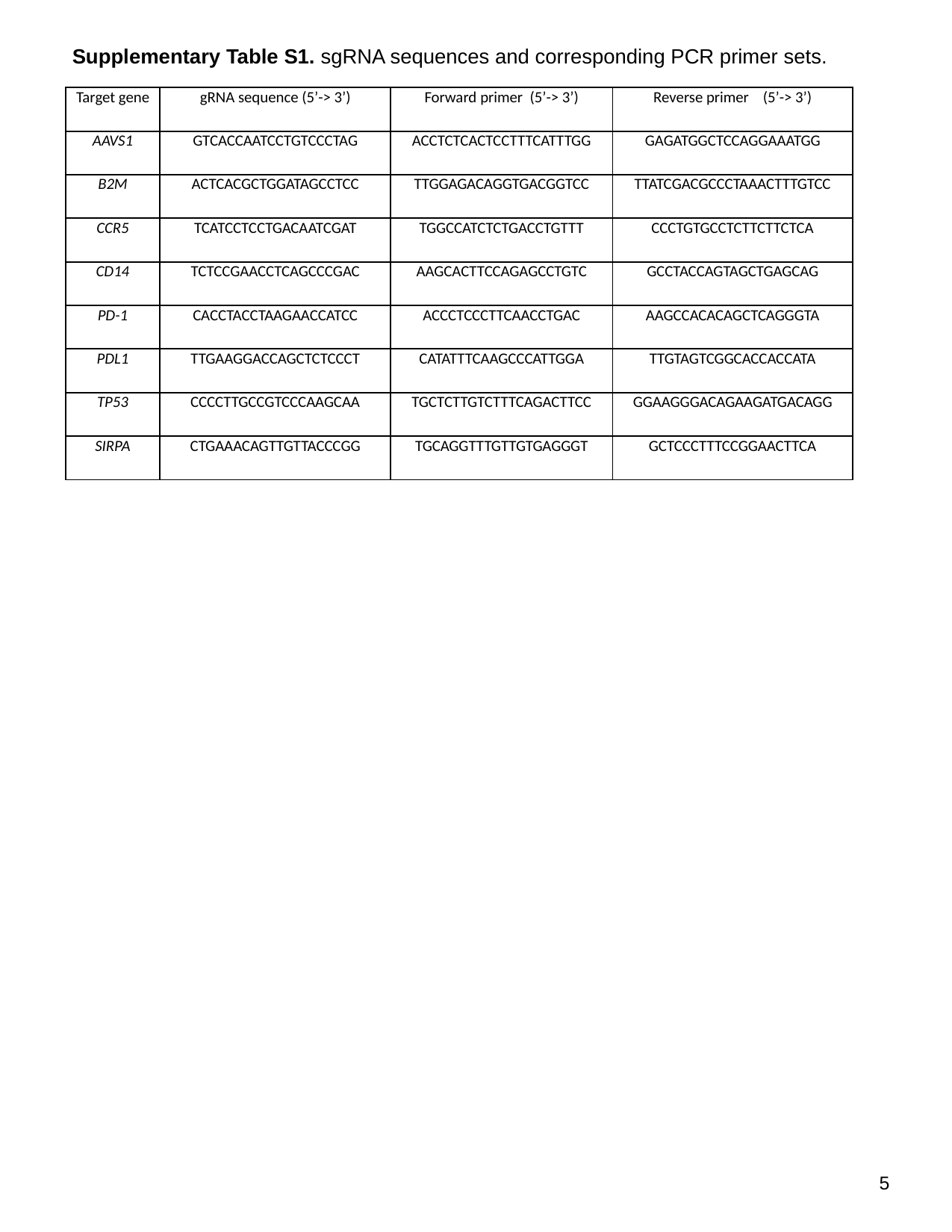

Supplementary Table S1. sgRNA sequences and corresponding PCR primer sets.
| Target gene | gRNA sequence (5’-> 3’) | Forward primer (5’-> 3’) | Reverse primer (5’-> 3’) |
| --- | --- | --- | --- |
| AAVS1 | GTCACCAATCCTGTCCCTAG | ACCTCTCACTCCTTTCATTTGG | GAGATGGCTCCAGGAAATGG |
| B2M | ACTCACGCTGGATAGCCTCC | TTGGAGACAGGTGACGGTCC | TTATCGACGCCCTAAACTTTGTCC |
| CCR5 | TCATCCTCCTGACAATCGAT | TGGCCATCTCTGACCTGTTT | CCCTGTGCCTCTTCTTCTCA |
| CD14 | TCTCCGAACCTCAGCCCGAC | AAGCACTTCCAGAGCCTGTC | GCCTACCAGTAGCTGAGCAG |
| PD-1 | CACCTACCTAAGAACCATCC | ACCCTCCCTTCAACCTGAC | AAGCCACACAGCTCAGGGTA |
| PDL1 | TTGAAGGACCAGCTCTCCCT | CATATTTCAAGCCCATTGGA | TTGTAGTCGGCACCACCATA |
| TP53 | CCCCTTGCCGTCCCAAGCAA | TGCTCTTGTCTTTCAGACTTCC | GGAAGGGACAGAAGATGACAGG |
| SIRPA | CTGAAACAGTTGTTACCCGG | TGCAGGTTTGTTGTGAGGGT | GCTCCCTTTCCGGAACTTCA |
5

## Slide 12
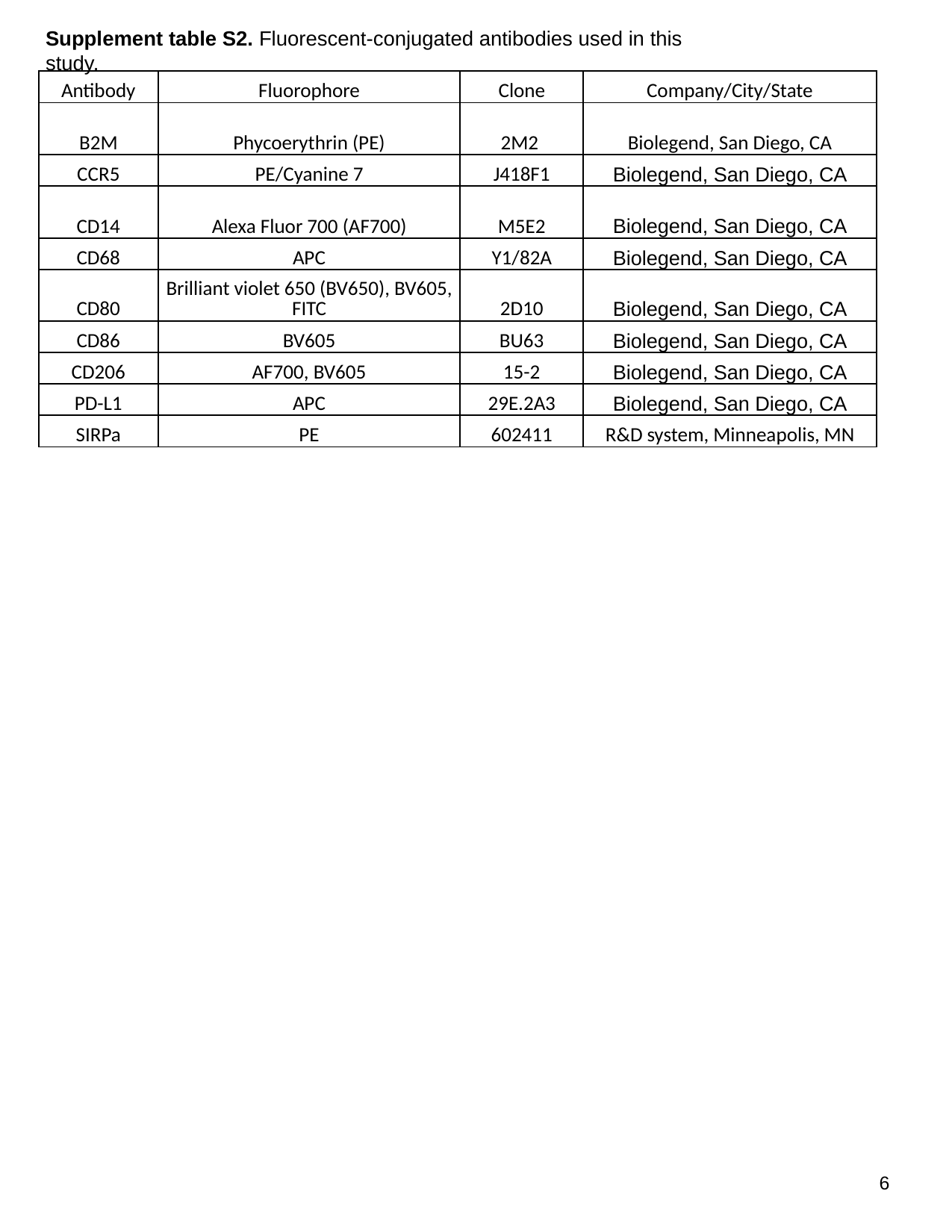

Supplement table S2. Fluorescent-conjugated antibodies used in this study.
| Antibody | Fluorophore | Clone | Company/City/State |
| --- | --- | --- | --- |
| B2M | Phycoerythrin (PE) | 2M2 | Biolegend, San Diego, CA |
| CCR5 | PE/Cyanine 7 | J418F1 | Biolegend, San Diego, CA |
| CD14 | Alexa Fluor 700 (AF700) | M5E2 | Biolegend, San Diego, CA |
| CD68 | APC | Y1/82A | Biolegend, San Diego, CA |
| CD80 | Brilliant violet 650 (BV650), BV605, FITC | 2D10 | Biolegend, San Diego, CA |
| CD86 | BV605 | BU63 | Biolegend, San Diego, CA |
| CD206 | AF700, BV605 | 15-2 | Biolegend, San Diego, CA |
| PD-L1 | APC | 29E.2A3 | Biolegend, San Diego, CA |
| SIRPa | PE | 602411 | R&D system, Minneapolis, MN |
6
